# A Game Theory-Based Model for Predicting Depression due to Frustration in Competitive Environments

**DOI:** 10.1155/2020/3573267

**Published:** 2020-06-03

**Authors:** R. Loula, L. H. A. Monteiro

**Affiliations:** ^1^Universidade Presbiteriana Mackenzie, PPGEEC, São Paulo SP, Brazil; ^2^Universidade de São Paulo, Escola Politécnica, São Paulo, SP, Brazil

## Abstract

A computational model based on game theory is here proposed to forecast the prevalence of depression caused by frustration in a competitive environment. This model comprises a spatially structured game, in which the individuals are socially connected. This game, which is equivalent to the well-known prisoner's dilemma, represents the payoffs that can be received by the individuals in the labor market. These individuals may or may not have invested in a formal academic education. It is assumed that an individual becomes depressed when the difference between the average payoff earned by the neighbors in this game and the personal payoff surpasses a critical number, which can be distinct for men and women. Thus, the transition to depression depends on two thresholds, whose values are tuned for the model accurately predicting the percentage of individuals that become depressed due to a frustrating payoff. Here, this tuning is performed by using data of young adults living in the United Kingdom in 2014-2016.

## 1. Introduction

Major depressive disorder, the mental illness popularly called depression, affects over 300 million people worldwide [1]. One relevant risk factor for depression is the lack of access to education [2]. Around 2010, in the United Kingdom, the access to higher education was facilitated [3]. However, in the following years, instead of decreasing, depression prevalence increased among men and women in the age range of graduating and starting to work [3–5]. Surveys conducted in 2014 [4] and 2016 [5] revealed that about 20% of young adults suffered from depression. Here, a computational model is proposed to investigate this issue.

The influence of job attributes (such as working conditions, workload, wage, stability, and career opportunities) on the occurrence of depression has been examined [6, 7]. Here, a negative perception of the career progress is considered a risk factor for depression. Thus, depression is supposed to be a consequence of unfulfilled expectations in the professional career. In the model, the insertion of young adults in their socioeconomic environment is represented by a game, which is shown to be equivalent to the prisoner's dilemma [8, 9].

Game theory has been employed in psychological studies, for instance, to analyze the influence of a depressive mood on economic decisions modeled by the ultimatum game [10] and to describe psychiatric disorders as Nash disequilibria of the self, reflecting discordances between actions and beliefs [11]. The game introduced here relates investment in formal education to professional success. It is supposed that, in a competitive environment, the individual becomes depressed when the difference between the average earning received by the neighbors in this game and the personal earning is above a threshold. Since this critical number can be different for men and women, then there are two thresholds ruling the transition to depression. The values of these parameters can be adjusted from real-world statistics, in order to improve the prediction accuracy of the model.

An individual-based probabilistic model was already proposed to forecast the prevalence of depression from a set of negative life events affecting a population, by taking into consideration its demographic and socioeconomic features [12]. Other approaches for detecting and predicting depression employ, for instance, artificial intelligence techniques [13–15] and signal processing tools [16–18]. Here, the model is formulated in terms of a game with spatial structure, a state transition rule (from healthy to sick), and a parameter adaptation algorithm.

This paper is organized as follows. In Section 2, the proposed model is introduced. In Section 3, the results obtained in computational simulations based on the data of young adults living in the United Kingdom in 2014-2016 are presented. In Section 4, the possible relevance of these results is discussed.

## 2. The Model

Consider a population of young adults (that is, individuals in the 18–24-year-old age range). Let this population be represented by a square lattice *n* × *n*, in which the top and bottom edges are connected and the right and left edges are also connected. This boundary condition is chosen to avoid edge effects. Each cell composing this toroidal lattice corresponds to an individual, who maintains social contact with the eight surrounding neighbors. In the literature on cellular automata, this regular coupling pattern is known as the Moore neighborhood of unit radius [19]. This regular graph has been employed, for instance, in works about the spreading of contagious diseases [20, 21]. The individuals of the proposed model have gender, in order to take into account that the number of women affected by depression is higher [22, 23]. Such individuals are randomly distributed over the lattice according to the gender statistics.

**Figure 1:**
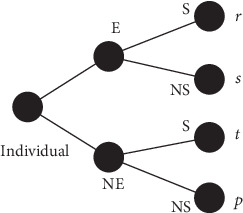
Extensive form representation of the investing-in-education game. In this game, E means academically educated (student), NE nonacademically educated (nonstudent), S success, and NS nonsuccess. The payoffs *t*, *r*, *p*, and *s* are received by the individual in function of his/her choice related to formal education (E or NE) and the corresponding performance in the labor market (S or NS).

Suppose that each individual had played once the investing-in-education game “against the field.” In a game against the field, there is no specific opponent. Here, the field is supposed to be the labor market. The investing-in-education game is summarized in Figure 1. The strategies that can be chosen by the individuals are E (academically educated, that is, student) and NE (nonacademically educated, that is, nonstudent). The strategy E is adopted by those who invest money and time in a standard academic education. The strategy NE is employed by those who do not care or do not have access to a formal education. The strategies provided by the field are S (success) and NS (nonsuccess). The strategy S means professional success (reaching a high socioeconomic status); NS means professional failure.

As shown in Figure 1, each individual receives one of the following payoffs: *t* for (NE,S), *r* for (E,S), *p* for (NE,NS), and *s* for (E,NS). Indeed, these payoffs are randomly distributed by the population, according to official statistics when available. For instance, if 10% of individuals have not had a formal education and did not achieve professional success, then the payoff *p* is randomly assigned to 10% of the population, regardless of the individual's gender.

It is reasonable to assume that success without investment in education is better than that with investment; therefore, *t* > *r*. Unsuccess without investment is better than that with investment; hence, *p* > *s*. Also, success with investment is better than unsuccess without investment; thus, *r* > *p*. In short, *t* > *r* > *p* > *s*. Note that these inequalities concerning the payoffs (with *r* > (*t* + *s*)/2) can also be found in the famous prisoner's dilemma [8, 9]. In this classic game theory example, two suspects are caught and accused of jointly committing a crime. When they are separately interrogated, their two options are D (defection) or C (cooperation). The strategy D means to confess to committing the crime; C means to remain silent. The payoff *t* corresponds to the temptation to defect against a cooperating partner, *r* is the reward for mutual cooperation, *p* is the punishment for mutual defection, and *s* is the sucker's payoff, which is the outcome of cooperating when the partner defects [8, 9]. The equivalence between the games can be established by replacing the individual and the field in the investing-in-education game by the two suspects involved in the prisoner's dilemma and by replacing the strategies E and S by C and the strategies NE and NS by D. Because of this equivalence, a rational individual who believes in destiny should not study (in this context, destiny is the lack of causal connection between formal education and success), since NE is the dominant strategy (giving the higher payoff) when the field plays either S or NS. However, people usually consider that investment in education is correlated with economic prosperity [24]. Notice that the mentioned equivalence is not perfect, since, in practice, the field does not (directly) receive any payoff; therefore, the investing-in-education game is not symmetric, as the prisoner's dilemma is.

To add variability, there are two kinds of individuals, who are denoted by A and B, in the proportions *c* and 1 − *c*, respectively. The payoffs earned by them are different when they are professionally successful. It is assumed that *t*_A_ > *t*_B_ and *r*_A_ > *r*_B_. Therefore, the parameters of this spatial game are the values of *t*_A_, *t*_B_, *r*_A_, *r*_*B*_, *p*, and *s* and the percentages of individuals who have gotten these payoffs.

Let *q*_*i*_ be the payoff earned by the *i*-th individual and $\bar{q}$ the average earning of the neighborhood (which is calculated from the values of *q*_*i*_ of the eight neighbors). All individuals are initially supposed to be healthy. The *i*-th individual becomes sick when:

1. $\bar{q}-q_{i}>\sigma^{*}$ for men
2. $\bar{q}-q_{i}>\tau^{*}$ for women

Observe that progression to depression occurs when the difference between the neighborhood payoff and the personal payoff is above a threshold, which can be different for men and women. Thus, the professional competence is subjectively assessed by comparing the wage in the current job to the neighbors' wages. A negative perception can lead to depression.

The thresholds *σ*^∗^ and *τ*^∗^ are tuned in the calibration phase. Assume that the variable *j* expresses the time steps of this phase. Let *x*(*j*) be the depression prevalence determined from the model at *j* and *x*_o_, the official estimate. From the initial values *σ*(0) and *τ*(0), *x*(0) is computed by initially taking *σ*^∗^ = *σ*(0) and *τ*^∗^ = *τ*(0). In the model, *ϵ* is the maximum absolute error allowed in the prediction of *x*_o_. Usually, it is necessary to adjust these two thresholds, in order to obtain such an error. The parameter adaptation algorithm is given by:

1. if *x*(*j*) − *x*_o_ > +*ϵ*, then *σ*(*j* + 1) = *σ*(*j*) − *∆* and *τ*(*j* + 1) = *τ*(*j*) − *∆*
2. if *x*(*j*) − *x*_o_ < −*ϵ*, then *σ*(*j* + 1) = *σ*(*j*) + *∆* and *τ*(*j* + 1) = *τ*(*j*) + *∆*

in which *∆* is a positive constant. When the official number *x*_o_ is achieved (with maximum error equal to *ϵ*), the ratio between the prevalence in women and in men is calculated. This ratio must be between 1.5 and 3.0 [4, 22, 23]. If it is outside this interval, then *∆* is subtracted from the current value of *τ* and a new step of the calibration phase is executed. The calibration finishes at the time step *T*, for which ∣*x*(*T*) − *x*_o_ | ≤*ϵ* and the ratio of sick women to sick men is between 1.5 and 3.0. Then, the threshold values are taken as *σ*^∗^ = *σ*(*T*) and *τ*^∗^ = *τ*(*T*), and *η* numerical simulations are performed in order to obtain $\bar{x}$, the average value of *x* in these *η* simulations.

In short, the simulation dynamics is the following. A gender and a payoff are randomly assigned (according to official statistics when available) to each individual of the lattice. He/she compares the average payoff of the neighborhood to his/her personal payoff. An individual becomes depressed if the difference between these payoffs is above a threshold. Since this threshold can be different for men and women, then, in fact, there are two thresholds, which are denoted by *τ*^∗^ and *σ*^∗^. These parameters are tuned in the calibration phase. The parameter adjustment ends when the target percentage of depressed individuals *x*_o_ and the expected ratio between the prevalence in women and in men are achieved. The values of *τ*^∗^ and *σ*^∗^ found in the calibration phase are assumed to be psychological characteristics of the studied population. Hence, after the calibration, the model can be used to determine, for instance, how the average prevalence $\bar{x}$ (obtained in *η* simulations) depends on the fractions of individuals receiving the six payoffs.

## 3. Numerical Results

Numerical simulations were performed with the payoffs *t*_A_ = 15, *t*_B_ = 10, *r*_A_ = 9, *r*_B_ = 6, *p* = 1, and *s* = −1. Also, *n* = 100 (therefore, the population is composed of 10000 individuals), *c* = 60% (thus, there are 60% of A individuals and 40% of B individuals), *ϵ* = 1% (the maximum absolute error), *∆* = 0.05 (the parameter used to tune *σ*^∗^ and *τ*^∗^ in the calibration phase), *η* = 100 (the number of simulations performed after finishing the calibration), and *σ*(0) = 1 and *τ*(0) = 1 (the initial values of the parameters ruling the transition to depression).

The target of the simulations is *x*_o_ = 20%, which is considered to be the prevalence of depression (due to personal frustration) in young adults in the United Kingdom in 2014-2016 [4, 5]. The gender distribution was 49.3% male and 50.7% female [25]. The percentage *ρ*_NE_ of nonstudents was 18.8% [26] (recall that nonstudents can receive the payoffs *t*_A_, *t*_B_, or *p*). Consequently, the percentage *ρ*_E_ of students was *ρ*_E_ = 1 − *ρ*_NE_ = 81.2% (and students can receive *r*_A_, *r*_B_, or *s*). Assume that *ρ*_NE,S_ = 5% is the percentage of successful nonstudents (who received *t*_A_ or *t*_B_) and *ρ*_E,S_ = 75% is the percentage of successful students (who received *r*_A_ or *r*_B_). With these choices, the prevalence predicted by the model was $\bar{x}=\left(19.94\pm 0.27\right)\%$, which is close to the target *x*_o_ = 20% [4, 5]. Also, *σ*^∗^ = 0.8 and *τ*^∗^ = 0.6, and the ratio between the prevalence in women and in men given by the model was 1.95, which belongs to the interval [1.5, 3.0].

**Figure 2:**
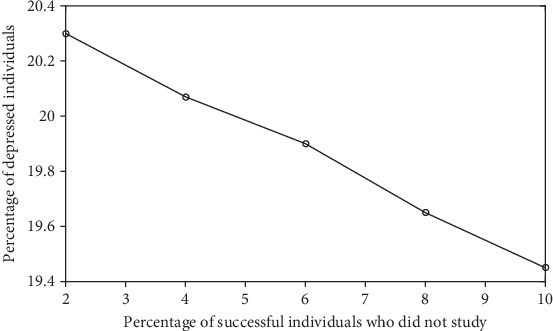
Average percentage $\bar{x}$ of depressed individuals in function of the percentage *ρ*_NE,S_ of successful nonstudents. The parameter values used in these simulations are *t*_A_ = 15, *t*_B_ = 10, *r*_A_ = 9, *r*_B_ = 6, *p* = 1, *s* = −1, *n* = 100, 49.3% male (and 50.7% female), *c* = 60%, *ϵ* = 1%, Δ = 0.05, *η* = 100, *σ*(0) = 1, *τ*(0) = 1, *x*_o_ = 20%, *ρ*_NE_ = 18.8%, and *ρ*_E,S_ = 75%.

**Figure 3:**
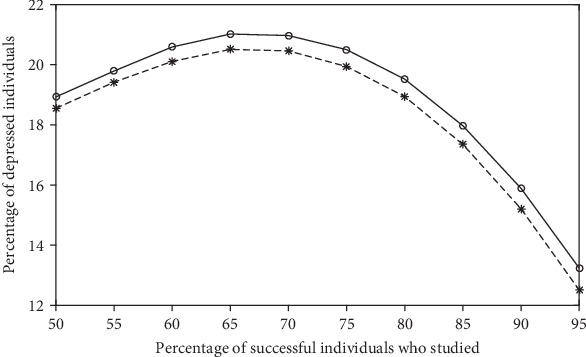
Average percentage $\bar{x}$ of depressed individuals in function of the percentage *ρ*_E,S_ of successful students for *ρ*_NE,S_ = 0% (solid line) and *ρ*_NE,S_ = 5% (dashed line). The other parameter values are the same as those used in Figure 1.

**Figure 4:**
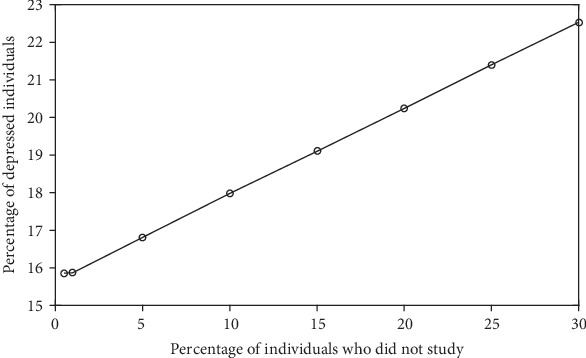
Average percentage $\bar{x}$ of depressed individuals in function of the percentage *ρ*_NE_ of nonstudents for *ρ*_E,S_ = 75% and *ρ*_NE,S_ = 5%. The other parameter values are the same as those used in Figure 1.

**Figure 5:**
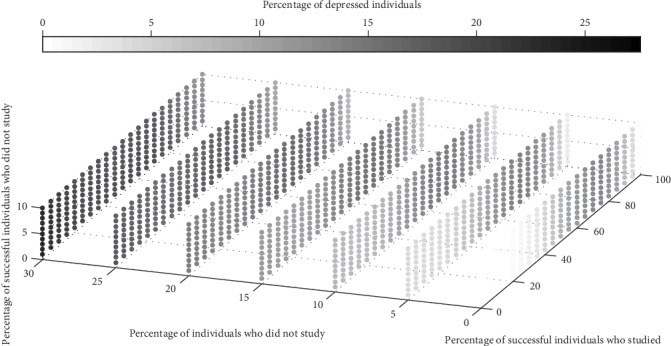
Average percentage $\bar{x}$ of depressed individuals in function of the percentages *ρ*_NE,S_ ∈ [0, 10%], *ρ*_NE_ ∈ [0, 30%], and *ρ*_E,S_ ∈ [0, 100%]. The other parameter values are the same as those used in Figure 1. The darker the circle, the higher the $\bar{x}$, as shown in the color bar.

In this first set of simulations, the percentages *ρ*_E,S_ and *ρ*_NE,S_ of successful individuals (who studied and who did not study, respectively) were arbitrarily chosen. To evaluate the impact of these percentages in the depression prevalence $\bar{x}$, other sets of simulations were run. These results are exhibited in Figures 2–5.

Figure 2 presents an expected outcome: $\bar{x}$ decreases by increasing *ρ*_NE,S_ (and consequently by reducing *ρ*_NE,NS_ in order to keep the percentage *ρ*_NE_ of nonstudents constant. Recall that *ρ*_NE_ = 18.8%). Thus, depression prevalence decreases by increasing the fraction of individuals receiving *t*_A_ or *t*_B_ (and concomitantly by reducing the fraction of individuals receiving *p*).

The plots shown in Figure 3 are surprising: the relation between $\bar{x}$ and *ρ*_E,S_ is not monotonous. The dashed line corresponds to *ρ*_NE,S_ = 5%, which can represent a socioeconomic environment. In the labor market, a small parcel of individuals does achieve professional success without investing in an academic formation (for instance, a digital influencer with a natural talent for humor or a very skilled soccer player). The solid line corresponds to *ρ*_NE,S_ = 0%, which can represent a learning environment. In a university, there is no academic achievement without studying. In both plots, the depression prevalence increases up to *ρ*_E,S_≃70% and, only above this number, $\bar{x}$ decreases (note that, as *ρ*_E,S_ increases, *ρ*_E,NS_ decreases, because *ρ*_E_ = 81.2% remains constant). Therefore, depression prevalence increases and then decreases by increasing the fraction of individuals receiving *r*_A_ or *r*_*B*_ (and concomitantly by reducing the fraction of individuals receiving *s*).

Figure 4 shows that the higher the *ρ*_NE_, the higher the $\bar{x}$. Thus, the higher the fraction of nonstudents, the higher the prevalence of depression.

The parameter values in Figures 2–4 are the same as those used in the first set of simulations. To get a full picture of this issue, additional sets of simulations were performed by varying the percentages *ρ*_E,S_, *ρ*_NE,S_, and *ρ*_NE_. In Figure 5, the darker the circle, the higher the $\bar{x}$. Observe that, for other values of *ρ*_NE,S_ and *ρ*_NE_ (different from those used in Figure 3), $\bar{x}$ has its maximum for intermediate values of *ρ*_E,S_.

## 4. Discussion and Conclusions

Depression due to social competition was already examined from an evolutionary point of view, through a discussion of the function of its mental state over the course of the human evolution [27]. Here, the occurrence of depression in competitive individuals was investigated from an educational-socioeconomic standpoint. It is undeniable that formal education promotes intellectual development, enhancing the opportunities in the labor market. However, many newly graduated students face unemployment and low occupational status; in other words, they have difficulties in finding a good job [3, 28, 29]. In fact, a higher education diploma favors, but does not guarantee, social progress. Also, analyzing the benefit-cost ratio concerning this investment can become a stressful experience, capable of triggering mental health problems, such as anxiety, hostility, and depression [3, 28, 29].

In the United Kingdom, depression prevalence increased in young adults, after the access to higher education being stimulated [3–5]. This observation can be explained by the proposed model, in which psychological distress emerges from comparisons of payoffs. Thus, a negative assessment of the personal success can lead to depression.

Figure 4 shows the expected relationship between the depression prevalence $\bar{x}$ and the percentage of nonstudents *ρ*_NE_; that is, $\bar{x}$ increases with *ρ*_NE_. The relationship between $\bar{x}$ and the percentage of successful nonstudents *ρ*_NE,S_ shown in Figure 2 was also not surprising; that is, $\bar{x}$ decreases with *ρ*_NE,S_. However, the dependence between $\bar{x}$ and the percentage of successful students *ρ*_E,S_ presented in Figure 3 is not obvious. Figure 5 reveals that this behavior can also be found for other parameter values.

As shown in Figure 3, $\bar{x}$ starts decreasing with *ρ*_E,S_ only above a critical number (about 70% in this figure). Therefore, from a government perspective, the access to higher education should be even more stimulated, in order to overcome this critical number. From an individual perspective, analysis of the benefit-cost ratio on investing in education should be avoided early in the career, because such a premature analysis can lead to wrong conclusions. In fact, the usual lack of emotional maturity of young adults can increase their vulnerability to stressful thoughts, like concerns about their jobs. Therefore, risk of depression can be reduced by setting realistic aspirations and by avoiding competitive comparisons of professional success.

## Data Availability

The data used to support the findings of this study are available from the corresponding author upon request.

## References

[1] World Health Organization (2017). *Depression and Other Common Mental Disorders: Global Health Estimates*.

[2] Licinio J., Wong M. L. (2005). *Biology of Depression: From Novel Insights to Therapeutic Strategies*.

[3] Cant S. (2018). Hysteresis, social congestion and debt: towards a sociology of mental health disorders in undergraduates. *Social Theory & Health*.

[4] McManus S., Bebbington P., Jenkins R., Brugha T. (2014). *Mental Health and Wellbeing in England: Adult Psychiatric Morbidity Survey*.

[5] Smith M. (2016). One in four students suffer from mental health problems. *YouGov*.

[6] Steyn R., Vawda N. (2014). Job characteristics: their relationship to job satisfaction, stress and depression. *Journal of Psychology in Africa*.

[7] Axelrod R. (2006). *The Evolution of Cooperation*.

[8] Szabó G., Fáth G. (2007). Evolutionary games on graphs. *Physics Reports*.

[9] Harlé K. M., Sanfey A. G. (2007). Incidental sadness biases social economic decisions in the ultimatum game. *Emotion*.

[10] Patokos T. (2011). The relevance of Nash equilibrium to psychiatric disorders. *Theoretical Medicine and Bioethics*.

[11] Loula R., Monteiro L. H. A. (2019). An individual-based model for predicting the prevalence of depression. *Ecological Complexity*.

[12] Mumtaz W., Ali S. S. A., Yasin M. A. M., Malik A. S. (2018). A machine learning framework involving EEG-based functional connectivity to diagnose major depressive disorder (MDD). *Medical & Biological Engineering & Computing*.

[13] Li X., La R., Wang Y. (2019). EEG-based mild depression recognition using convolutional neural network. *Medical & Biological Engineering & Computing*.

[14] Akar S. A., Kara S., Agambayev S., Bilgiç V. (2015). Nonlinear analysis of EEGs of patients with major depression during different emotional states. *Computers in Biology and Medicine*.

[15] Jiang H., Hu B., Liu Z. (2018). Detecting depression using an ensemble logistic regression model based on multiple speech features. *Computational and Mathematical Methods in Medicine*.

[16] Bachmann M., Päeske L., Kalev K. (2018). Methods for classifying depression in single channel EEG using linear and nonlinear signal analysis. *Computer Methods and Programs in Biomedicine*.

[17] Wolfram S. (1994). *Cellular Automata and Complexity: Collected Papers*.

[18] Dias J. C. A., Monteiro L. H. A. (2018). Clustered Breeding Sites: Shelters for Vector-Borne Diseases. *Computational and Mathematical Methods in Medicine*.

[19] Ferraz D. F., Monteiro L. H. A. (2019). The impact of imported cases on the persistence of contagious diseases. *Ecological Complexity*.

[20] American Psychiatric Association (2013). *Diagnostic and Statistical Manual of Mental Disorders*.

[21] Marginson S. (2016). The worldwide trend to high participation higher education: dynamics of social stratification in inclusive systems. *Higher Education*.

[22] Office for National Statistics (2017). *Population by age, gender and ethnicity*.

[23] Offce for National Statistics (2019). Measuring national well-being in the UK: international comparisons. *ONS*.

[24] Jackson P. B., Finney M. (2002). Negative life events and psychological distress among young adults. *Social Psychology Quarterly*.

[25] Grenfell M. (2014). *Pierre Bourdieu: Key Concepts*.

[26] Zimmerman F. J., Christakis D. A., Vander Stoep A. (2004). Tinker, tailor, soldier, patient: work attributes and depression disparities among young adults. *Social Science & Medicine*.

[27] Lee Y., Ragguett R. M., Mansur R. B. (2018). Applications of machine learning algorithms to predict therapeutic outcomes in depression: a metaanalysis and systematic review. *Journal of Affective Disorders*.

[28] Piccinelli M., Gomez Homen F. (1997). *Gender Differences in the Epidemiology of Affective Disorders and Schizophrenia*.

[29] Price J., Sloman L., Gardner R., Gilbert P., Rohde P. (1994). The social competition hypothesis of depression. *British Journal of Psychiatry*.

